# Using data from a multi-hospital clinical network to explore prevalence of pediatric rickets in Kenya

**DOI:** 10.12688/wellcomeopenres.12038.2

**Published:** 2017-11-01

**Authors:** Stella W. Karuri, Maureen K. Murithi, Grace Irimu, Mike English, Clinical Information Network authors

**Affiliations:** 1 Kenya Medical Research Institute-Wellcome Trust Research Programme, Nairobi, Kenya; 2 Nuffield Department of Clinical Medicine, University of Oxford, Oxford, OX3 7BN, UK

**Keywords:** Rickets, Hospital Records, Pediatric Nutrition, Hospital Stays, Kenya, Malnutrition, Pneumonia

## Abstract

**Background:** Nutritional rickets is a public health concern in developing countries despite tropical climates and a re-emerging issue in developed countries. In this study, we reviewed pediatric admission data from the Clinical Information Network (CIN) to help determine hospital and region based prevalence of rickets in three regions of Kenya (Central Kenya, Western Kenya and Nairobi County). We also examine the association of rickets with other diagnosis, such as malnutrition and pneumonia, and study the effect of rickets on regional hospital stays.

**Methods:** We analyzed discharge records for children aged 1 month to 5 years from county (formerly district) hospitals in the CIN, with admissions from February 1 ^st^ 2014 to February 28 ^th^ 2015. The strength of the association between rickets and key demographic factors, as well as with malnutrition and pneumonia, was assessed using odds ratios. The Fisher exact test was used to test the significance of the estimated odd ratios. Kaplan-Meier curves were used to analyze length of hospital stays.

**Results:** There was a marked difference in prevalence across the three regions, with Nairobi having the highest number of cases of rickets at a proportion of 4.01%, followed by Central Region at 0.92%. Out of 9756 admissions in the Western Region, there was only one diagnosis of rickets. Malnutrition was associated with rickets; this association varied regionally. Pneumonia was found to be associated with rickets in Central Kenya. Children diagnosed with rickets had longer hospital stays, even when cases of malnutrition and pneumonia were excluded in the analysis.

**Conclusion:** There was marked regional variation in hospital based prevalence of rickets, but in some regions it is a common clinical diagnosis suggesting the need for targeted public health interventions. Factors such as maternal and child nutrition, urbanization and cultural practices might explain these differences.

## Abbreviations

CIN, Clinical Information Network; FET, Fisher Exact Test; KEMRI, Kenya Medical Research Institute; LOS, length of stay; LRT, likelihood ratio test; OR, odds ratio; WAZ, weight for age Z-score

## Introduction

Prolonged vitamin D deficiency causes rickets, the softening and weakening of bones due to inadequate mineralization, and which leads to bone deformities. Rickets has been documented in at least 59 countries in the last 20 years ^1^ and is a pediatric concern, particularly in developing countries ^2^. Rickets is an emerging concern in developed countries ^3^. Vitamin D deficiency occurs in diets low in calcium, vitamin D or phosphates ^4, 5^. Vitamin D is also synthesized when human skin is exposed to sunlight; dark skin synthesizes vitamin D at a slower rate ^6^. Therefore, children in low income sub-Saharan Africa countries are perhaps paradoxically some of the most susceptible to rickets ^7, 8^.

In Kenya, anecdotal evidence suggests considerable variability in the prevalence of childhood rickets, but no published data support this, and rickets is not generally considered a major public health problem. We therefore sought to explore the prevalence of rickets in contrasting settings in Kenya, using data from the Clinical Information Network (CIN). The CIN is a collaborative initiative between The Kenya Pediatric Association, The Ministry of Health and the KEMRI-Wellcome Trust Research Programme (KWTRP). The network includes thirteen county hospitals from across Kenya and works to support better data collection on hospitalized children ^9^. CIN hospitals provide first referral services at county (formerly district) level, and were purposefully selected to have representation from high and low malaria endemic areas ^10^. Some network hospitals serve populations that are entirely urban, but most serve mixed rural/urban catchment populations. In addition, we explore the association between rickets and demographic risk factors such as age, gender and nutrition status. We explore the association of rickets with co-morbidities, such as pneumonia and malnutrition, before discussing possible factors that could explain the regional variation of pediatric rickets.

## Methods

This was an exploratory study using data collected within the CIN from February 1 ^st^ 2014 to February 28 ^th^ 2015. The network encouraged hospitals to implement two data collection tools: a pediatric admission record form and a discharge form. Data collection was conducted as soon as possible after discharge through abstracting data from inpatient paper records into a non-propriety electronic tool, according to detailed standard operating procedures and with in-built range and validity checks. Additional error correction procedures were employed locally and centrally in the CIN. The methods for data collection have been described in full elsewhere ^9^. No identifiable data were collected; each of the records was given a unique study identification number at the time of data entry to maintain patient confidentiality. Data required for the national reporting system was collected for each pediatric admission in each hospital and more comprehensive data on disease specific care processes, including investigations and treatment, were collected in all acute medical admissions (excluding neonates) in 10 low to moderate workload hospitals, and on a random subset of similar medical admissions in three high workload hospitals.

For the purposes of this study, we divided hospitals in the CIN into three groups based on their location. A full description of these hospitals’ patient populations can be found elsewhere ^10^.

- Nairobi County – H3 and H4 serving a city population,
- Central Kenya – H1, H2, H5, H6, H7 and H14 in the highland or semi-arid central part of Kenya,
- Western Kenya – H8, H9, H10, H11 and H12 in the western malaria endemic are of Kenya.

Our case definition of rickets was any discharge diagnosis in the hospital records of ‘rickets’. This diagnosis is made at the discretion of the clinical team and typically based on clinical identification of frontal bossing, wrist cupping, rachitic rosary, or lower limb deformities pathognomonic for rickets. X-rays might sometimes be used in the diagnosis, but such supporting evidence was not required to identify cases in this study. Other diagnoses used in the study were clinical diagnoses and were based on recommended national ^11^ and WHO guidelines ^12^, encoded by ICD-10. For these analyses, we studied admitted children aged between 1 month and 5 years.

R version 3.1.3 was used for analysis. Descriptive statistics were used to summarize population demographics. Variables were summarized with means, medians and standard deviations, counts and proportions as appropriate. Associations between categorical variables were represented with odds-ratios (OR). The strength of association was assessed with the Fisher Exact Test (FET), with significant associations considered as having p-value ≤0.05. A time-to-event grouped analysis of the length of stay (LOS) using Kaplan-Meier probabilities was also performed. Deaths were treated as right censored observations in this analysis. The log-rank test (LRT) was used to compare LOS in subgroups of interest, significantly different LOS between groups were declared for p-value ≤0.05. An adjusted analysis for rickets (adjusting for all demographic factors) was not numerically feasible because there were an insufficient number of rickets incidences. Furthermore, a regional adjustment was not possible because Western Region had approximately zero rickets cases.

## Results

The total number of eligible hospital admissions for these analyses was 20,528 with a total of 9,756 admissions in hospitals in Western Kenya. Table 1 gives site specific summary statistics on key demographic variables. There were slightly more male admissions than female. Pooling data from all sites, the mean weight was approximately 10 kilograms (95% CI: 9.9 – 10.0), while the mean age was approximately 21.4 months (95% CI: 21.2 – 21.6). The proportion of admissions to a site with weight for age Z (WAZ) score < -2 ranged from 14% to 41%, with the sites in Nairobi County having the highest proportions. Approximately 75% admissions had a LOS less than 5 days. There were 1,188 deaths, which made up 5.4% of all admissions. The deaths in the Nairobi, Central and Western Regions make up 11%, 3.3% and 6.7%, respectively of the admissions in the study period.

**Table 1.** Summary statistics for demographic characteristics by site. Q1=first quantile, Q3=third quantile, SE=standard error.

Region	Hospital	N = 20528	Male (%)	WAZ < -2 (%)	Mean age, months (SE)	Died (%)	LOS median (Q1: Q3)
Nairobi	H3	1198	671 (56.01)	376 (33.84)	16.04 (13.19)	112 (9.69)	3 ( 1 : 5 )
	H4	1493	818 (54.79)	573 (40.58)	17.13 (14.27)	163 (12.22)	5 ( 3 : 9 )
Central	H5	1508	844 (55.97)	331 (22.55)	19.97 (14.02)	43 (2.87)	3 ( 2 : 4 )
	H14	1923	1125 (58.5)	552 (30.07)	18.61 (14.06)	87 (4.58)	3 ( 2 : 6 )
	H1	1077	591 (54.87)	272 (26.05)	16.02 (13.33)	9 (0.87)	3 ( 2 : 5 )
	H7	1383	760 (54.95)	254 (18.53)	21.06 (14.73)	31 (2.27)	2 ( 1 : 5 )
	H6	1060	560 (52.83)	190 (20.72)	20.11 (14.19)	30 (2.90)	4 ( 2 : 6 )
	H2	1130	640 (56.64)	260 (26.89)	18.14 (14.55)	68 (6.05)	4 ( 2 : 6 )
Western	H11	1881	1049 (55.77)	353 (19.83)	23.76 (15.94)	125 (6.72)	2 ( 1 : 4 )
	H9	2888	1520 (52.63)	455 (17.96)	26.11 (17.09)	209 (7.29)	3 ( 2 : 4 )
	H12	2124	1157 (54.47)	318 (15.47)	21.47 (16.06)	134 (6.46)	3 ( 2 : 5 )
	H8	1592	887 (55.72)	204 (15.84)	25.10 (16.71)	108 (6.95)	2 ( 2 : 4 )
	H10	1271	703 (55.31)	169 (14.20)	26.81 (16.34)	69 (5.47)	2 ( 1 : 3 )

**Figure 1.**
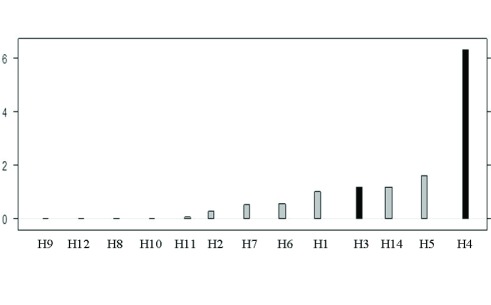
Site specific prevalence of rickets. White for Western sites, grey for Central sites, black for Nairobi sites.

Figure 1 is a bar plot of the site specific prevalence of rickets. There was only one case of rickets in the entire Western Region. This one case does not provide sufficient counts for meaningful inference on the association of rickets with other factors of interest; therefore the Western Region was excluded in any association analysis. The highest proportion of rickets cases (6% admissions) was in H4. Table 2 gives regional frequencies and proportion of rickets cases by age, gender, WAZ scores and LOS sub-groups. Statistical evidence from the data indicates that in the Nairobi and Central Regions, rickets occurs predominantly in younger children (age ≤2 years) and in children with WAZ <-2. The results also indicate that children with rickets are likely to have had longer hospital stays.

**Table 2.** Regional odds-ratios and FET p-values for association between rickets and age, gender, nutrition status (WAZ <-2) and LOS >2 subgroups. LOS=length of stay, WAZ= weight for age Z-score, (*) indicates Fisher Exact Test p-value ≤ 0.05.

Nairobi					
Factor	With rickets (N=108)		Without rickets (N=2583)		Odds-ratio (p-value)
	N	(%)	N	(%)	
Age ≤24 months	102	(94.44)*	2044	(79.13)	4.48 (<0.01)
Male gender	62	(57.41)	1427	(55.25)	1.09 (0.70)
WAZ <-2	69	(66.35)*	880	(36.38)	3.45 (<0.01)
LOS >2 days	66	(89.19)*	1656	(64.11)	6.16 (<0.01)
Central					
Factor	With rickets (N=74)		Without rickets (N=8007)		Odds-ratiobreak/>(p-value)
	**N**	**(%)**	**N**	**(%)**	
Age <= 24 months	72	(97.3)*	5809	(72.55)	13.62 (<0.01)
Male gender	42	(56.76)	4478	(55.93)	1.03 (0.91)
WAZ < -2	52	(72.22)*	1807	(23.99)	8.23 (<0.01)
LOS > 2 days	99	(91.67)*	4789	(59.81)	5.54 (<0.01)

Table 3 presents analysis of the association between rickets and the clinical diagnosis of pneumonia, as well as the association of rickets and the clinical diagnosis of severe malnutrition. Severe malnutrition was classified as any of the following diagnoses: Kwashiorkor [ICD10: E40], Marasmus [ICD10: E41], Severe Malnutrition [ICD10: E43] or Marasmus-Kwashiorkor [ICD10: E42]. The proportions of admissions diagnosed with severe malnutrition in the Nairobi, Central and Western Region are 10%, 6% and 3%, respectively. There is evidence of an association between clinical diagnoses of severe malnutrition and rickets; the odds of children diagnosed with severe malnutrition also being diagnosed with rickets increase by a factor of 2 in Nairobi and a factor of 9 in Central province. The proportions of admissions diagnosed with pneumonia in the Nairobi, Central and Western Regions are 56%, 54% and 32%, respectively. The only significant association of pneumonia and rickets occurs in the Central Region; the odds of a rickets diagnosis in addition to a pneumonia diagnosis increases by a factor of 1.7.

**Table 3.** Regional association of rickets with diagnoses of severe malnutrition and pneumonia.

Nairobi					
	Without rickets (N = 2583)		With rickets (N = 108)		Odds-ratio (p-value)
	N	%	N	%	
Severe malnutrition	251	(9.72)	21	(19.44)	2.24 (<0.01)
Pneumonia	1520	(58.85)	67	(62.04)	1.14 (0.55)
Central					
	Without rickets (N = 8007)		With rickets (N = 74)		Odds-ratio (p-value)
	N	%	N	%	
Severe malnutrition	465	(5.81)	27	(36.49)	9.31 (<0.01)
Pneumonia	4924	(61.5)	54	(72.97)	1.69 (0.05)

**Figure 2.**
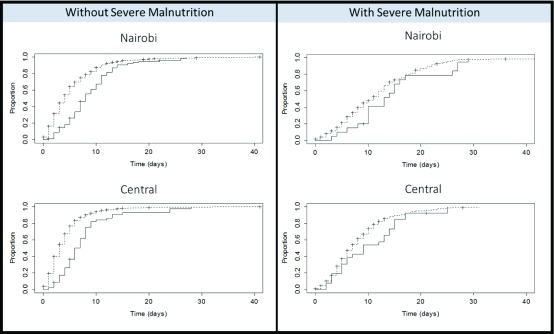
Regional proportions of patients discharged within given times stratified by absence/presence of severe malnutrition. Solid line, diagnosed with rickets; dashed line, not diagnosed with rickets.

**Figure 3.**
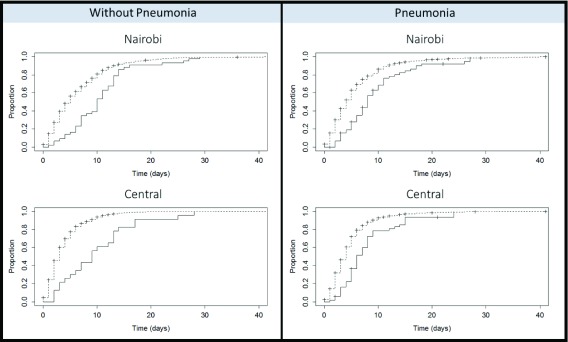
Regional proportions of patients discharged within given times stratified by absence/presence of pneumonia. Solid line, diagnosed with rickets; dashed line, not diagnosed with rickets.

Figure 2 and Figure 3 are plots of the proportion of children discharged with a LOS of at most *T* days, where *T* is given by the x-axis. The analysis is grouped by region (Nairobi and Central) and presence or absence of a clinical diagnosis of either pneumonia or severe malnutrition. Among children without severe malnutrition, children diagnosed with rickets were more likely to have longer hospital stays (LRT p-value <0.01 in both the Nairobi and Central Regions), while among children diagnosed with severe malnutrition, there was no association between rickets and LOS (LRT p-value of 0.13 and 0.10 for the Nairobi and Central Regions, respectively). Among children who were diagnosed with pneumonia, children with rickets tended to have a longer LOS (LRT p-values <0.01 for both Nairobi and Central Regions), even when children diagnosed with severe malnutrition were excluded from analysis (LRT p-values <0.01 for both Nairobi and Central Regions).

Table 4 is a study of the association between rickets and death for the Central and Nairobi regions, among children with clinical diagnoses of pneumonia, severe malnutrition, malnutrition (including milder forms of malnutrition), dehydration or diarrhea. There is no evidence suggesting that the presence of rickets was associated with increased mortality in the listed diagnosis groups. Furthermore, in admissions diagnosed with malnutrition (including milder forms of malnutrition), there was no evidence of an association between mortality and rickets.

**Table 4.** Association between death and rickets in diagnosis groups for Central and Nairobi Regions. P-values from the Fisher Exact Test.

	Pneumonia (n=5709)	
	Without rickets	With rickets
	n=5600	n=109
Deaths n (%)	345 (6.16)	9 (8.26)
*Odds-ratio*	*1.37 (p-value = 0.32)*	
	Severe malnutrition (n=727)	
	Without rickets	With rickets
	n=680	n=47
Deaths n (%)	88 (12.94)	2 (4.26)
*Odds-ratio*	*0.30 (p-value = 0.11)*	
	Malnutrition (Severe, moderate, mild) (n=1150)	
	Without rickets	With rickets
	n=1079	n=71
Deaths n (%)	118 (10.94)	3 (4.23)
*Odds-ratio*	*0.36 (p-value = 0.11)*	
	Dehydration (n=2233)	
	Without rickets	With rickets
	n=2180	n=53
Deaths n (%)	230 (10.55)	4 (7.55)
*Odds-ratio*	*0.69 (p-value = 0.65)*	
	Diarrhea, n=2805	
	Without Rickets	With Rickets
	n=2772	n=33
Deaths n (%)	172 (6.2)	2 (6.06)
*Odds-ratio*	*0.97 (p-value=1.00)*	

## Discussion

Rickets has been linked to protein energy malnutrition ^13, 14^ and respiratory infections in developing countries ^15^. Our study reveals marked variation in the prevalence of rickets in different regions in Kenya. Where rickets occurs, it is strongly associated with a clinical diagnosis of severe malnutrition and shows some association with a diagnosis of pneumonia in one region; the strength of these associations also exhibits regional variation. Evidence from our data also indicates that children with rickets have longer hospital stays, even in the subgroup made up of cases where diagnoses of severe malnutrition or pneumonia are excluded. However, we cannot be sure whether this increased LOS is linked to poorer recovery from co-morbid conditions or a consequence of retaining the child to treat rickets.

Poverty, which is associated with rickets ^16^, might explain the observed regional variation in rickets prevalence. Recent evidence suggest that clinical diagnoses of pediatric rickets are common in a slum area in Nairobi ^17^ and are linked to vitamin D deficiency ^18^. The percentage of adults living in extreme poverty in the counties with hospitals’ in the CIN ranges from 21% to 65% ^10^. Using these crude population estimates of poverty provides no clear picture: Nairobi has moderately high levels of poverty and the highest prevalence of rickets, while overall levels of poverty are as high in the Western region with almost no rickets. Furthermore, poverty levels are at their lowest in the Central Region with some rickets. Given that actual populations using hospitals may vary considerably this may not be surprising. Public hospitals in the Nairobi region are likely to serve a disproportionate number of patients from the densely populated slum areas, while the poorest people may struggle to access hospital care in more rural Western Kenyan settings.

Inability to access nutrient rich food ^19^ and nutritional diversity are other possible explanations for the regional effect. Communities in Western Kenya have both vegetable and fish based diets, with nutrient-rich indigenous vegetables, such as leaves of amaranth grains ( *Amaranthus* sp.) widely consumed. The fish *Dagaa* ( *Rastrineobola argentea*), which is high in micro-nutrient density (calcium and vitamin D), fatty acids and proteins, is widely consumed and is commonly sifted and incorporated into weaning flours ^20^. Historically, the diet for communities in Central Kenya is mostly cereal based ^21, 22^, with little diversity in the diet ^23^. The practice of early weaning to cereal porridge by communities in Central Kenya is thought to deprive infant’s proteins and micronutrients found in human milk ^24^, which predisposes infants to mineral deficiencies, such as rickets and anemia.

Cultural practices and weather might also influence regional prevalence of rickets. Swaddling of babies has been associated with rickets ^25, 26^. Swaddling is practiced in the colder regions of Central Kenya, where the average temperature for the coldest month in some locations, such as Nyeri town, is 10°C. Western Kenya, however, is considerably warmer; for example, the average temperatures for the coldest month in Kisumu, a major Western Kenya town, is 22°C. Children here are often minimally clad, hence experience more sun exposure.

A limitation of our work is that rickets in most cases is likely to be diagnosed and treated as an outpatient condition, and only in severe cases, or when a serious comorbid illness is present, are such cases admitted. Our data may therefore suggest what the pattern of rickets is, but cannot be used to infer the possible magnitude of vitamin D deficiency in children. In addition, our analysis is based on inpatient data from routine pediatric admission records with no prospective sensitization or training of clinicians in diagnosing rickets, and in settings where routine diagnostic tests are rarely available ^10^. These factors may result in the under-estimation of the true hospital based prevalence or, if some areas have been sensitized to the problem of rickets, may contribute to apparent regional differences. Furthermore, causal relationships cannot be ascertained. Nonetheless, these limitations are unlikely to affect the main conclusion of our study, the low rates of rickets in Western Kenya compared to Central Kenya and Nairobi. The results of this study are useful in that they suggest the need for more robust studies to help obtain good quality data that would more accurately depict the scale of the problem of vitamin D deficiency and rickets, better define regional variation and help identify local risk factors. With prevalence of rickets as high as 6% of admissions in some areas, such studies would appear urgent and targeted public health measures may be needed to avert long term consequences of an entirely preventable dietary deficiency. Such measures might include: improved use of calcium or vitamin D supplementation in pregnant and lactating women, infant supplementation or food fortification.

Given the known problems associated with the sensitivity and the specificity of a clinical diagnosis of nutritional rickets, there is value in hospital based prospective studies for better estimation of prevalence rates and subtle Vitamin D deficiency. Well-designed prospective studies should feature a standardized definition of a clinical diagnosis of nutritional rickets. These studies should also support the use of routine wrist X-rays, with scans reviewed by trained radiologists for a confirmatory diagnosis. In addition, a rickets diagnosis should be supported by more definitive biochemical testing.

## Data availability

The data referenced by this article are under copyright with the following copyright statement: Copyright: © 2017 Karuri SW et al.

The source data are owned by the Kenyan Ministry of Health, County Governments and individual county hospitals and the study authors are not permitted to share the source data that supports the Clinical Information Network. As the Kenyan Ministry of Health does not have the data in aggregate form, and this is held by individual licensed facilities, users who wish to reuse the source data have to begin a request initially through the KEMRI-Wellcome Trust Research Programme data governance committee. This committee will supply contact information for the KEMRI Scientific and Ethical Review unit and the Kenyan Ministry of Health as appropriate. The KEMRI-Wellcome Trust Research Programme data governance committee can be contacted on: dgc@kemri-wellcome.org.
