# Supplementary material for: Using data from a multi-hospital clinical network to explore prevalence of pediatric rickets in Kenya
Source: Wellcome Open Res. 2017 Nov 1;2:64. Originally published 2017 Aug 17. [Version 2] doi: 10.12688/wellcomeopenres.12038.2 (PMC5629544; doi:10.12688/wellcomeopenres.12038.2)
Supplement: Peer review history

# Peer review: Referee response for version 1

**DOI:** 10.21956/wellcomeopenres.13024.r25860

**Published:** 2017-10-05

**Authors:** Kelsey D. J. Jones

**Affiliations:** 1 Chelsea & Westminster Hospitals NHS Foundation Trust, London, UK

This is a well-written paper on a neglected health problem in Kenya. Two recent studies have contributed anecdotal data that suggest a significant burden of disease in urban areas, and have identified vitamin D deficiency as the likely cause of rickets in many cases (note that this is different to most other settings in Africa). The current study provides the first proper epidemiologic data from Kenya, and there are some remarkable findings. That 6% of paediatric inpatients in a hospital in a region with abundant sunlight have rickets is quite extraordinary; could the authors put this into context - has similar prevalence been recorded elsewhere?  

 In general the approaches and analyses are sound. There is a lot of emphasis on length of stay, I presume as a proxy for severity/complexity, though it is a difficult parameter to interpret (certainly in low-resource settings in Kenya, where length of stay can be artificially inflated by delays in payment, for example). I don’t feel that figures 2 or 3 add a great deal to the argument and I would be minded put them into a supplementary file.

 Two frequently reported associations of vitamin D deficiency are developmental delay and TB. Can the authors look for any association with these conditions? I expect that developmental delay is probably not well coded in your data so it may not be possible, but I bet that TB is coded much better. I think this would really add value to the paper. (A section will need to be added to the discussion about the difficulty in robustly diagnosing TB in Kenya and similar settings.)

 The discussion section is sound, but would do well to highlight even further the difficulty in diagnosing rickets in Kenya and similar settings, which makes studying it so difficult. Rickets is best diagnosed by a combination of clinical, radiographical and biochemical features, but I expect that most children in this study were diagnosed clinically alone. Clinical evaluation is insensitive – especially where the clinician is concentrating on ‘urgent’, severe illness requiring hospital admission, but it can also lack specificity: anecdotally, in Nairobi I have come across children diagnosed with rickets solely on the basis of neurodevelopmental delay, for which there are a multitude of other potential causes.  The authors could provide specific guidance on the kind of prospective clinical research that would help to resolve theses difficult issues.

 NB1: Source data is not provided but I would not expect it to be for a study of this type due to practical and ethical issues.

 NB2: The authors might like to consider citing the two recent articles on rickets in Nairobi that have helped to highlight it as a public health concern ^1^^-^^2^ I declare a conflict of interest in this suggestion as I am an author on one of these papers, but I think that it strengthens their argument because it provides evidence that vitamin D deficiency usually underlies rickets in Nairobi - an assumption the authors make throughout their article.

I have read this submission. I believe that I have an appropriate level of expertise to confirm that it is of an acceptable scientific standard.

## References

[1] JonesKDJHachmeisterCUKhasiraMCoxLSchoenmakersIMunyiCNassirHSHünten-KirschBPrenticeABerkleyJA: Vitamin D deficiency causes rickets in an urban informal settlement in Kenya and is associated with malnutrition. *Matern Child Nutr*.2017; 10.1111/mcn.12452 10.1111/mcn.12452 28470840PMC5763407

[2] EdwardsJKThiongóAVan den BerghRKizitoWKosgeiRJSobryAVandenbulckeAZunigaIReidAJ: Preventable but neglected: rickets in an informal settlement, Nairobi, Kenya. *Public Health Action*.2014;4(2) : 10.5588/pha.14.0009 122-7 10.5588/pha.14.0009 26399212PMC4539038

# Response

**Published:** 2017-10-29

**Authors:** Stella Karuri

**Affiliations:** Kenya Medical Research Institute, Kenya

We thank the reviewer for the review and the comments. Our response to each of the comments is given in italics below:

This is a well-written paper on a neglected health problem in Kenya. Two recent studies have contributed anecdotal data that suggest a significant burden of disease in urban areas, and have identified vitamin D deficiency as the likely cause of rickets in many cases (note that this is different to most other settings in Africa). The current study provides the first proper epidemiologic data from Kenya, and there are some remarkable findings. That 6% of paediatric inpatients in a hospital in a region with abundant sunlight have rickets is quite extraordinary; could the authors put this into context - has similar prevalence been recorded elsewhere?  

*Review articles by Prentice ^1^ and more recently by Creo et al ^2^ cite studies in areas with abundant sunshine in Asia and Africa where prevalence of nutritional rickets was estimated to be higher than 6%. For example, a nationwide survey estimated a prevalence of 8% in children under 10 years of age ^3^ and a survey study in Northern Nigeria estimated a prevalence of 9% in children under 3 years of age ^4^.*

In general the approaches and analyses are sound. There is a lot of emphasis on length of stay, I presume as a proxy for severity/complexity, though it is a difficult parameter to interpret (certainly in low-resource settings in Kenya, where length of stay can be artificially inflated by delays in payment, for example). I don’t feel that figures 2 or 3 add a great deal to the argument and I would be minded put them into a supplementary file.

*We understand the reviewer’s point of view, however we believe that the length-of-stay (LOS) metric suggest a cost aspect to a rickets diagnosis. It is rare for patients to be detained for non-payment of hospital fees in the sites in the study. Furthermore the discharge dates used in computing LOS were the medical discharge date and not the administrative discharge date. There is also a potential, at least for pneumonia, that clinical signs such as lower chest wall indrawing take longer to resolve in children with Rickets resulting in longer stays.*

Two frequently reported associations of vitamin D deficiency are developmental delay and TB. Can the authors look for any association with these conditions? I expect that developmental delay is probably not well coded in your data so it may not be possible, but I bet that TB is coded much better. I think this would really add value to the paper. (A section will need to be added to the discussion about the difficulty in robustly diagnosing TB in Kenya and similar settings.)

*We did explore the relationship between TB and rickets however the admission and discharge data in our network was not rich enough for robust inference, we had less than 1% of admissions with a TB diagnosis which were mostly clinical diagnoses. This rate is likely un under-diagnosis of TB, diagnosing TB in young children is difficult. Advanced TB diagnostics tools such as GeneXpert have not yet entered common use in the sites in our study.  It is widely felt that there is under-diagnosis of TB in younger children and specific work is being undertaken to make better diagnostic tools available with a view to exploring the links of TB with Vitamin D deficiency.*

The discussion section is sound, but would do well to highlight even further the difficulty in diagnosing rickets in Kenya and similar settings, which makes studying it so difficult. Rickets is best diagnosed by a combination of clinical, radiographical and biochemical features, but I expect that most children in this study were diagnosed clinically alone. Clinical evaluation is insensitive – especially where the clinician is concentrating on ‘urgent’, severe illness requiring hospital admission, but it can also lack specificity: anecdotally, in Nairobi I have come across children diagnosed with rickets solely on the basis of neurodevelopmental delay, for which there are a multitude of other potential causes.  The authors could provide specific guidance on the kind of prospective clinical research that would help to resolve theses difficult issues.

*We have added a concluding paragraph to the text addressing this comment. Please see the updated version of the article. We are also grateful to the reviewer for bringing to our attention these new studies of rickets and have cited them in the new version.*

***References***

 

1. *Prentice, A. (2013). Nutritional rickets around the world. The Journal of steroid biochemistry and molecular biology, 136, 201-206.*
2. *Creo, A. L., Thacher, T. D., Pettifor, J. M., Strand, M. A., & Fischer, P. R. (2017) . Nutritional rickets around the world: an update. Paediatrics and international child health, 37(2), 84-98.*
3. *(Craviari, T., Pettifor, J. M., Thacher, T. D., Meisner, C., Arnaud, J., Fischer, P. R., & Group, R. C. (2008). Rickets: An Overview and Future Directions, with Special Reference to Bangladesh: A Summary of the Rickets Convergence Group Meeting, Dhaka, 26–27 January 2006. Journal of health, population, and nutrition, 26(1), 112.*
4. *(Heaney, R. P., Abrams, S., Dawson-Hughes, B., Looker, A., Marcus, R., Matkovic, V., & Weaver, C. (2000). Peak bone mass. Osteoporosis international, 11(12), 985-1009.)*

# Peer review: Referee response for version 1

**DOI:** 10.21956/wellcomeopenres.13024.r25222

**Published:** 2017-09-04

**Authors:** Nicholas Shaw

**Affiliations:** 1 Department of Endocrinology & Diabetes, Birmingham Children's Hospital, Birmingham, UK; 2 Institute of Metabolism and Systems Research, University of Birmingham, Birmingham, UK

This article presents relevant and useful information about the prevalence of nutritional rickets in 3 different regions of Kenya reflecting a mixture of urban and rural populations. Utilizing hospital admission and discharge data on over 20,000 admissions routinely collected through the Clinical Information Network they were able to identify children under 5 years where "rickets" was recorded in the discharge diagnosis. Significant differences were noted across the three regions with only one case identified in the Western region with the highest prevalence reported from one hospital in Nairobi.

 The majority of cases as would be anticipated were under the age of 2 years with a significant association with malnutrition defined as a weight for age Z-score (<-2.0). Previous work has suggested an association between rickets and/or vitamin D deficiency with pneumonia. However despite a high prevalence of admissions with pneumonia such an association was only seen in the Western region. It is also of interest that no increased risk of mortality was seen in children with rickets given the potential association between vitamin D deficiency and innate immunity.

 The authors discuss potential explanations for the variation in prevalence seen across the three regions such as rates of poverty particularly in Nairobi. They have highlighted the potential relevance of differences in dietary practices in particular the lack of dietary calcium intake of the cereal based diet in Central Kenya. It is now well recognized that dietary calcium intake has an important role in addition to vitamin D deficiency in the aetiology of nutritional rickets.

 The authors have highlighted the potential limitations of their work particularly the fact that many children with rickets are managed as outpatients and therefore will not be identified in hospital discharge data. Another important factor is the dependence on clinical signs such as lower limb deformities for the diagnosis of rickets. The recent Global Consensus guidelines on Nutritional Rickets ^1^ recommended that the diagnosis is made on the basis of history,physical examination and biochemical results and is confirmed by radiographs. It is recognized that access to such investigations may be difficult in countries such as Kenya but it would have been useful if radiographs were available in a subset of the sample to see what proportion of cases of rickets were confirmed on X-ray. In a similar setting in the Gambia where bone deformity was used as a diagnostic marker for rickets only in 9% of cases was this confirmed by X-ray ^2^.

 Two additional comments I would make are:

 1) Considering the survey was conducted over 1 year it would be useful if the authors were able to calculate the incidence of nutritional rickets assuming they have information on the population of children less than 5 years in the regions surveyed. Incidence data for this condition is quite limited worldwide.

 2) It is not clear to this reviewer what is represented on the Y-axis of Figure 2.

I have read this submission. I believe that I have an appropriate level of expertise to confirm that it is of an acceptable scientific standard.

## References

[1] MunnsCFShawNKielyMSpeckerBLThacherTDOzonoKMichigamiTTiosanoDMughalMZMäkitieORamos-AbadLWardLDiMeglioLAAtapattuNCassinelliHBraeggerCPettiforJMSethAIdrisHWBhatiaVFuJGoldbergGSävendahlLKhadgawatRPludowskiPMaddockJHyppönenEOduwoleAFrewEAguiarMTulchinskyTButlerGHöglerW: Global Consensus Recommendations on Prevention and Management of Nutritional Rickets. *J Clin Endocrinol Metab*.2016;101(2) : 10.1210/jc.2015-2175 394-415 10.1210/jc.2015-2175 26745253PMC4880117

[2] JonesHLJammehLOwensSFulfordAJMooreSEPettiforJMPrenticeA: Prevalence of rickets-like bone deformities in rural Gambian children. *Bone*.2015;77: 10.1016/j.bone.2015.04.011 1-5 10.1016/j.bone.2015.04.011 25871880PMC4456426
